# Packaging methods and storage duration affect essential oil content and composition of lemon verbena (*Lippia citriodora* Kunth.)

**DOI:** 10.1002/fsn3.434

**Published:** 2016-10-19

**Authors:** Mohammad‐Taghi Ebadi, Fatemeh Sefidkon, Majid Azizi, Noorollah Ahmadi

**Affiliations:** ^1^ Department of Horticultural SciencesFaculty of AgricultureTarbiat Modares UniversityTehranIran; ^2^ Research Institute of Forests and RangelandsTehranIran; ^3^ Department of Horticultural SciencesFaculty of AgricultureFerdowsi University of MashhadMashhadIran

**Keywords:** Lemon verbena (*Lippia citriodora* Kunth.), essential oil, packaging, storage, citral

## Abstract

Changes in essential oils (EOs) content and composition of lemon verbena leave at different packaging methods (packaged with air, nitrogen, or under vacuum) and during storage period (0, 2, 4, 6 and 8 months) were determined. All the samples were hydrodistilled every 2 months during storage for EO content evaluation. EO composition was determined by gas chromatography and gas chromatography–mass spectrometry. The results showed that by extending the storage period in all packaging methods, EO content was significantly decreased. Parallel to the increase in the storage duration in all packaging methods, citral content was decreased, whereas the amounts of limonene and 1,8‐cineole were increased. Packaging of lemon verbena leaves with nitrogen preserved the highest EO content during 8 months of storage and achieved the desired amounts of citral, limonene, and 1,8‐cineole. This investigation also showed camphene may be a useful marker for the indication of storage duration of lemon verbena.

## 1 Introduction

The genus *Lippia* consists of approximately 200 species of shrubs, herbs, or trees which belong to the family Verbenaceae (Argyropoulou, Akoumianaki‐Ioannidou, Christodoulakis, & Fasseas, 2010). They are largely distributed throughout South and Central America and Tropical Africa (Pascuala, Slowing, Carretero, Sanchez Mata, & Villar, 2001). *Lippia citriodora* Kunth. (syn. *Lippia triphylla* Kuntze; *Aloysia triphylla* Britton) is cultivated mainly for the lemon‐like aroma emitted from its leaves which are utilized for the preparation of herbal tea (Argyropoulou et al., 2010; Carnat, Carnat, Fraisse, & Lamaison, 1999). *L. citriodora* is utilized as a gastrointestinal and respiratory remedy in traditional medicine (Pascuala et al., 2001). Also, lemon verbena has a long history of folk uses in treating colds, fever, insomnia, and anxiety (Carnat et al., 1999).

Medicinal and aromatic plants (MAPs) are often stored for long periods before use, in order to manufacture various types of products (Mahmoodi Sourestani, Malekzadeh, & Tava, 2014). MAPs undergo many physical, chemical, and microbiological changes during storage (Mayuoni‐Kirshinbaum, Daus, & Porat, 2013; Peter, 2006). The protective coating provided during processing, storage, and handling not only retards deterioration of MAPs but may also improves their quality (Pääkkönen, Malmsten, & Hyvönen, 1990; Peter, 2006). Packaging fulfills several purposes, including preventing contamination during distribution, preserving product integrity, and maintaining the desired flavor profile of the product (Chaliha, Cusack, Currie, Sultanbawa, & Smyth, 2013). Unsuitable packaging and storage can induce alterations in the chemical composition of their active substance which may affect the flavor and fragrance properties of the herbal product, with a negative impact on the industrial value and consumer satisfaction (Potisate, Kerr, & Phoungchandang, 2015; Rosado, Pinto, Bertolucci, Jesus, & Alves, 2013). Therefore, determining a suitable packaging method to maintain higher concentrations of active substances during storage is very important and some researchers have paid attention to this topic, for example: Lin, Sung, and Chen (2011) reported that packing material affected the storability of coneflower (*Echinacea purpurea* L.) materials. They showed dried plant in polyethylene terephthalate/aluminum foil/polyethylene or nylon/polyethylene bags and stored under low temperature without light retained the highest content of bioactive compounds. Orav, Stulova, Kailas, and Müürisepp (2004) observed that after 1 year of storage of pepper (*Piper nigrum* L.) in a glass vessel at room temperature, the amount of the oil and terpenes decreased, but the amount of oxygenated terpenoids increased. Pääkkönen, Malmsten, and Hyvönen (1989) reported that the intensity of odor and taste of dill (*Anethum graveolens* L.) in 1 year storage were better preserved in vacuum packages than in glass jars or paper bags. Rosado et al. (2013) observed no significant differences in essential oils (EO) content of basil (*Ocimum basilicum* L.) stored in paper or plastic bags over a 12‐month period; but, independent of packaging, the oil content was decreased by 0.1% per month. Pääkkönen et al. (1990) reported the intensity scores of odor and flavor of summer savory (*Satureja hortensis* L.) samples were packed into polyethylene–aluminum–polyethylene bags under vacuum and in nitrogen atmosphere were significantly higher than those of the samples stored in glass jars and paper bags after 9 months of storage. Díaz‐Maroto, Pardo, Castillo‐Muñoz, Díaz‐Maroto, and Pérez‐Coello (2009) observed that rosemary (*Rosmarinus officinalis* L.) samples packaged in polystyrene bottles retained a higher quantity of volatile compounds than those packaged in glass bottles after 21 months of storage.

Although the effects of packaging and storage on the quality of MAPs have been studied in research studies mentioned, little information regarding the effects of these processes on *L. citriodora* exists and this is a topic of great interest for both industry and consumer. The aim of this research was to investigate the effect of packaging methods and storage duration on the EO content and composition of *L. citriodora*.

## 2 Materials and Methods

### 2.1 Plant material

Fresh leaves of lemon verbena (*Lippia citriodora* Kunth.) used in this research were harvested from the greenhouse of the Department of Horticulture, Tarbiat Modares University (TMU), Iran. The samples were shade dried for 3 days at room temperature (20–25°C).

### 2.2 Packaging and storage

In the laboratory, lemon verbena leaves were packed under vacuum, nitrogen, and air gas in polyamide/polyethylene laminate bags (size 35×25 cm, thickness 50 μm; Chap Iran Zamin Co. Ltd., Iran), by using a packaging machine (Henkelman 200A, The Netherlands). Also, identical samples were placed in boxes without cover as controls. The average weight of the leaves in each package was 15 g. The samples of all treatments were stored at 25±3°C and 35±5% RH for 8 months and every 2 months, sampling was done for EOs analysis. In addition, to determine the exact effects of storage conditions on EOs compositions during the experimental period, one sample of leaves was analyzed at the start of the experiment.

### 2.3 EO isolation

The EOs of all samples were extracted by hydrodistillation using a Clevenger‐type apparatus. Fifteen grams of leaves from each sample was placed in a round‐bottomed flask containing 300 ml of distilled water. Distillation was continued for approximately 3 hr and the EO content was determined on the basis of dry matter (ml/100 g D.M.). The EOs were dried over anhydrous sodium sulfate to eliminate traces of moisture and stored in tightly closed dark vials at 4°C until analysis.

### 2.4 Gas chromatography

The EOs were analyzed by Gas chromatography (GC), using a Thermo‐UFM ultra‐fast gas chromatograph equipped with a Ph‐5‐fused silica column (10 m×0.1 mm i.d., film thickness 0.4 μm). The oven temperature was held at 60°C for 5 min and then programmed to 285°C at a rate of 80°C/min. Detector (FID) temperature was 280°C and the injector temperature was 280°C; helium was used as carrier gas with a linear velocity of 0.5 ml/min. The percentages of compounds were calculated by the area normalization method, without considering response factors.

### 2.5 Gas chromatography–mass spectrometry

Gas chromatography–mass spectrometry (GC‐MS) analyses were performed using a Varian 3400 GC‐MS system equipped with a DB‐5‐fused silica column (30 m×0.25 mm i.d., film thickness 0.25 μm); oven temperature was 50–240°C at a rate of 4°C/min, transfer line temperature 260°C, carrier gas helium with a linear velocity of 31.5 cm/s, split ratio 1:60, ionization energy 70 eV, scan time 1 s, and mass range 40–300 a.m.u. The EO components were identified by comparison of their mass spectra with those of a computer library or with authentic compounds and confirmed by comparison of their retention indices, either with those of authentic compounds or with data published in the literature (Adams, 2007; Davies, 1990). Mass spectra from the literature were also compared (Adams, 2007). The retention indices were calculated for all volatile constituents, using a homologous series of n‐alkanes.

### 2.6 Data analysis

This research was conducted using a factorial experiment based on completely randomized design with two factors (method of packaging and storage duration) and three replicates. Differences in means were tested by using a Duncan's Multiple Range Test (SAS) at 5% level of significance.

## 3 Results and Discussion

### 3.1 Effect of packaging methods and storage duration on EO content

The results of the EO content analysis in different packaging methods and storage duration are presented in Table 1. The results showed that packaging methods and storage duration had a significant effect on EO content (*p*<.05). By extending storage period in all packaging methods, EO content was significantly decreased. For example, EO content in air and under vacuum packed samples decreased 36.4 and 27.3%, respectively, after 8 months of storage. The highest reduction in EO content (45%) was observed in the control treatment. Packaging with nitrogen preserved EO content better than other methods and no significant difference was observed dependent upon duration storage. The EO content evolution following 2, 4, 6, and 8 months after storage in nitrogen packed samples was 1.1, 1.0, 0.95, and 0.95 ml/100 g D.M., respectively. Nitrogen, an inert gas, is often the preferred gas in cases where the aim of gas packaging is to protect the plants from undesirable oxidative changes (Phillips, 1996). In addition, other researchers have reported positive effects of packaging with nitrogen on the quality of MAPs (Pääkkönen et al., 1989). The loss of EOs during storage has been reported in several MAPs. For example, Díaz‐Maroto et al. (2009) observed a large loss of volatile compounds from rosemary in the first 6 months of storage. Baritaux, Richard, Touche, and Derbesy (1992) reported that the losses of EO content were 19%, 62%, and 66% in 3, 6, and 7 months storage of basil, respectively. Mahmoodi Sourestani et al. (2014) reported that the EO yield of anise hyssop (*Agastache foeniculum* (Pursh.) Kuntze.) was affected mainly by storage time and decreased about 0.6% after 2 months. Also, Martinazzo et al. (2009) in lemon grass (*Cymbopogon citratus* Stapf.) and Arabhosseini, Huisman, Boxtel, IIer, and Mu (2007) in tarragon (*Artemisia dracunculus* L.) reported a reduction in the EO content during storage. This phenomenon may be due to evaporation and oxidative reactions (Baritaux et al., 1992; Mockute, Bernotiene, & Judpentiene, 2005; Rowshan, Bahmanzadegan, & Saharkhiz, 2013).

**Table 1:** EO content (V/W %) of lemon verbena after treated by different packaging methods and storage duration

Packaging method	Storage duration (month)				
	0	2	4	6	8
Control	1.1±0.08^a^	1.0±0.07^ab^	0.8±0.04^cd^	0.7±0.03^de^	0.6±0.015^e^
Air	1.1±0.1^a^	1.0±0.05^ab^	0.9±0.03^bc^	0.8±0.02^cd^	0.7±0.03^de^
Nitrogen	1.1±0.09^a^	1.1±0.08^a^	1.0±0.05^ab^	0.95±0.05^abc^	0.95±0.03^abc^
Vacuum	1.1±0.09^a^	1.1±0.1^a^	1.0±0.04^ab^	0.9±0.02^bc^	0.8±0.04^cd^

Mean values followed by different letters in each row are significantly different at *p*<.05.

### 3.2 Effect of packaging methods and storage duration on EO composition

**Figure 1:**
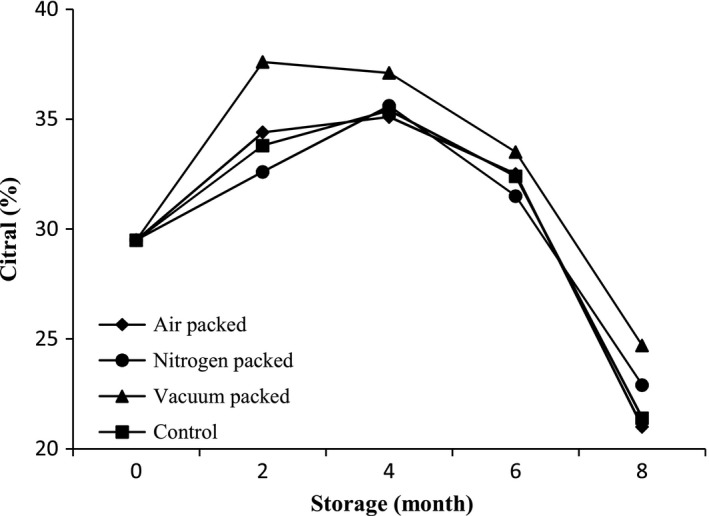
Citral content (%) of lemon verbena in different packaging methods and storage duration

In this experiment, 24 compounds were identified in the EOs of *L. citriodora* as being affected by different packaging methods and storage duration; that which represented 91.7–98.2% of the EOs present. The chemical compounds of the EOs have been presented in Tables 2, 3, 4, and 5. The main components of the EO in all treatments were geranial, neral, limonene, and 1,8‐cineole. Also, γ‐elemene, spathulenol, and globulol were determined between 5 and 10%. The results showed that packaging methods and storage duration caused some variation in the main components of EO (geranial, neral, limonene, and 1,8‐cineole) and altered the chemical profiles of lemon verbena EO. In all the packages with increasing storage duration up to 4 months, the amount of citral (geranial+neral) initially increased and then gradually decreased to the eighth month (Fig. 1). The reduction in citral under vacuum packaging happened more slowly than other methods and packaging with air and control treatment had the greatest reduction. At the beginning of the experiment, the amount of citral in EO was 29.5%, however, after 8 months of storage, citral content in packaging with air, nitrogen, and under vacuum was determined at 21, 22.9, and 24.7%, respectively. In the control treatment, the amount of citral was observed to be 21.4% at the end of the experiment. The amount of limonene was increased in all packaging methods during 8 months of storage and this compound was increased 30.5, 37.2, 27.1, and 32.5% in packaging with air, nitrogen, under vacuum, and control treatment, respectively. 1,8‐cineole was increased similar to limonene and this increase was 48.4, 45.9, 42.9, and 49.4% in packaging with air, nitrogen, vacuum and control treatment, respectively. In the case of γ‐elemene and globulol, although the amount of these was reduced at the end of the storage period, fluctuations were observed. The lowest reduction in these compounds was observed under vacuum packaging and they were decreased by 8.6 and 29.1%, respectively. Compounds such as γ‐terpinene, terpinolene, and α‐humulene were increased during storage in all packaging methods, but spathulenol, E‐caryophyllene, and epi‐α‐cadinol were decreased (Tables 2, 3, 4, and 5). Changes in the essential components affected by packaging methods and storage duration have been reported by several other researchers. For example, Pääkkönen et al. (1989) reported when storage was at room temperature, the organoleptic properties (correlated with EO components) of dill in 12 months storage were better preserved under vacuum packages than in glass jars or paper bags. Sakamura (1987) observed an increase in nerol and geraniol concentrations and a reduction in geranial acetate during storage of ginger (*Zingiber officinale* Roscoe.). He considered the conversion of geranyl acetate into geraniol, geranial, and neral, successively. Rosado et al. (2013) reported that the relative concentrations of the major constituents of basil, linalool and geraniol were 76.1% and 16.7%, respectively, for leaves stored in paper bags for 12 months and 77.1% and 16.6%, respectively, for leaves stored in plastic bags. Chaliha et al. (2013) observed that the packaging with PET/PET/Foil/PE bags in lemon myrtle (*Backhousia citriodora* F. Muell.) caused the highest amount of key volatiles neral and geranial at the end of 6 months of storage, but for anise myrtle (*Syzygium anisatum* Craven & Biffen.) and Tasmanian pepper (*Tasmannia lanceolata* A.C.Sm.) leaves, there was no significant difference between samples stored in PET/PET/Foil/PE bags and those stored in PVDC‐coated PET/CPP bags in the retention of key volatiles. Baritaux et al. (1992) reported that the amount of methylchavicol and eugenol decreased during 7 months of storage of basil, but the content of linalol and 1,8‐cineole increased. The reasons for these changes may be due to the water activity or available water in the product, availability of oxygen, and presence of temperature during storage. For MAPs, the most important factors in preserving quality are water and oxygen transmission rates (Chaliha et al., 2013). Also, the change in the EO composition during storage depends on the type of compound, the plant species, and the storage conditions (Mahmoodi Sourestani et al., 2014). Moreover, certain volatile compounds such as citral can migrate into the packaging material, which will produce changes in levels found (Chaliha et al., 2013). Misharina (2001) imagined terpene biotransformation as a reason for these changes. He explained: terpenes are able to bind or release a water molecule, to isomerize or rearrange, and EO components themselves, or trace contaminants, may catalyze or initiate these reactions. It has been observed that the composition of EOs readily changes upon processing and storage, whereby factors such as temperature, light, and oxygen availability have a crucial impact on alteration processes (Díaz‐Maroto et al., 2009).

**Table 2:** EO constituents (%) of air packed lemon verbena in storage duration

No.	Compound	RI^a^	Storage duration (month)				
			0	2	4	6	8
1	α‐pinene	938	0.4	0.5	0.4	t	t
2	Camphene	953	t	t	t	0.4	0.8
3	Sabinene	981	1.0	1.5	1.0	1.1	2.0
4	Limonene	1028	13.7	14.8	14.7	12.5	19.7
5	1,8‐cineole	1031	13.3	11.1	15.7	12.0	25.8
6	γ‐terpinene	1062	0.8	0.8	1.2	1.6	2.0
7	Terpinolene	1090	0.6	0.4	0.8	1.2	1.4
8	Transpinocarveol	1140	t	t	t	0.7	t
9	cis‐sabinol	1143	t	t	t	t	0.1
10	Citronellal	1152	0.5	0.8	0.6	0.3	0.4
11	α‐terpineol	1190	1.2	1.5	1.4	2.3	1.4
12	Nerol	1228	0.4	0.4	0.4	0.3	t
13	Neral	1238	11.7	13.9	13.2	13.2	7.8
14	Geranial	1267	17.8	20.5	21.9	19.3	13.2
15	Neryl acetate	1360	1.0	0.8	1.1	1.3	0.7
16	α‐copaene	1379	0.6	0.5	0.6	0.7	0.6
17	α‐gurjunene	1410	t	t	t	t	t
18	E‐caryophyllene	1421	0.5	0.9	0.9	0.7	0.6
19	γ‐elemene	1439	8.1	7.5	8.2	9.1	7.0
20	α‐humulene	1456	1.0	1.0	1.8	1.9	1.8
21	Cubenol	1514	0.4	0.4	0.6	0.9	0.6
22	Spathulenol	1580	10.2	6.1	5.1	4.8	4.7
23	Globulol	1587	8.1	7.4	6.3	9.4	5.5
24	Epi‐α‐cadinol	1642	1.2	0.9	0.6	0.8	0.5
Monoterpene hydrocarbons		18.1	16.5	18	18.1	16.8	25.9
Oxygenated monoterpenes		54.3	45.9	49	54.3	49.4	49.4
Sesquiterpene hydrocarbons		11.5	10.2	9.9	11.5	12.4	10
Oxygenated sesquiterpenes		12.6	19.9	14.8	12.6	15.9	11.3
Total		96.5	92.5	91.7	96.5	94.5	96.6

a RI, retention indices relative to C8–C25 n‐alkanes on the DB‐5 column; t, trace <0.1%.

**Table 3:** EO constituents (%) of nitrogen packed lemon verbena in storage duration

No.	Compound	RI^a^	Storage duration (month)				
			0	2	4	6	8
1	α‐pinene	938	0.4	0.7	0.5	t	t
2	Camphene	953	t	t	0.3	0.3	0.9
3	Sabinene	981	1.0	1.6	2.4	1.0	1.4
4	Limonene	1028	13.7	15.6	16.7	12.4	21.8
5	1,8‐cineole	1031	13.3	10.5	15.4	13.0	24.6
6	γ‐terpinene	1062	0.8	0.7	0.8	1.5	2.0
7	Terpinolene	1090	0.6	0.3	0.8	1.2	1.5
8	Transpinocarveol	1140	t	t	0.3	t	t
9	cis‐sabinol	1143	t	t	0.2	0.7	t
10	Citronellal	1152	0.5	0.6	1.0	0.3	0.5
11	α‐terpineol	1190	1.2	1.3	1.4	2.2	1.4
12	Nerol	1228	0.4	0.3	0.3	0.4	t
13	Neral	1238	11.7	12.5	14.3	13.1	8.6
14	Geranial	1267	17.8	20.1	21.3	18.4	14.3
15	Neryl acetate	1360	1.0	2.0	0.9	1.1	0.6
16	α‐copaene	1379	0.6	0.5	0.5	0.7	0.5
17	α‐gurjunene	1410	t	1.0	t	t	t
18	E‐caryophyllene	1421	0.5	1.2	0.8	0.7	0.6
19	γ‐elemene	1439	8.1	7.0	7.0	8.7	6.8
20	α‐humulene	1456	1.0	0.6	1.6	1.9	1.7
21	Cubenol	1514	0.4	1.0	0.5	0.3	0.5
22	Spathulenol	1580	10.2	6.4	4.2	4.5	3.7
23	Globulol	1587	8.1	8.6	5.7	9.9	4.9
24	epi‐α‐cadinol	1642	1.2	1.0	0.5	0.9	0.4
Monoterpene hydrocarbons			16.5	18.9	21.5	16.4	27.6
Oxygenated monoterpenes			45.9	47.3	55.1	49.2	50
Sesquiterpene hydrocarbons			10.2	10.3	9.9	12	9.6
Oxygenated sesquiterpene			19.9	17	10.9	15.6	9.5
Total			92.5	93.5	97.4	93.2	96.7

a RI, retention indices relative to C8–C25 n‐alkanes on the DB‐5 column; t: trace <0.1%.

**Table 4:** EO constituents (%) of vacuum packed lemon verbena in storage duration

No.	Compound	RI^a^	Storage duration (month)				
			0	2	4	6	8
1	α‐pinene	938	0.4	0.3	0.3	t	t
2	Camphene	953	t	t	0.4	0.6	0.8
3	Sabinene	981	1.0	1.2	2.0	1.5	1.2
4	Limonene	1028	13.7	12.3	13.4	18.5	18.8
5	1,8‐cineole	1031	13.3	8.8	12.7	7.2	23.3
6	γ‐terpinene	1062	0.8	0.6	0.8	1.5	2.0
7	Terpinolene	1090	0.6	0.3	0.7	1.1	1.4
8	Transpinocarveol	1140	t	t	t	t	t
9	cis‐sabinol	1143	t	t	t	0.5	t
10	Citronellal	1152	0.5	t	0.5	0.2	0.5
11	α‐terpineol	1190	1.2	1.3	1.4	2.1	1.4
12	Nerol	1228	0.4	0.3	0.4	0.4	t
13	Neral	1238	11.7	13.5	15.2	13.5	9.1
14	Geranial	1267	17.8	24.1	21.9	20.0	15.6
15	Neryl acetate	1360	1.0	1.3	1.0	1.4	0.8
16	α‐copaene	1379	0.6	t	0.6	0.6	0.6
17	α‐gurjunene	1410	t	t	0.2	0.3	t
18	E‐caryophyllene	1421	0.5	3.6	0.9	0.8	0.6
19	γ‐elemene	1439	8.1	5.6	8.5	8.2	7.4
20	α‐humulene	1456	1.0	0.5	1.7	1.7	2.0
21	Cubenol	1514	0.4	2.4	0.8	1.0	0.9
22	Spathulenol	1580	10.2	5.9	5.4	6.4	4.5
23	Globulol	1587	8.1	7.7	7.3	10.2	5.6
24	Epi‐α‐cadinol	1642	1.2	1.1	0.5	0.5	0.5
Monoterpene hydrocarbons			16.5	14.7	17.6	26.2	24.2
Oxygenated monoterpenes			45.9	49.3	53.1	38.1	50.7
Sesquiterpene hydrocarbons			10.2	10.6	11.9	11.6	10.6
Oxygenated sesquiterpenes			19.9	17.1	14	18.1	11.5
Total			92.5	91.7	96.6	98.2	97

a RI, retention indices relative to C8–C25 n‐alkanes on the DB‐5 column; t, trace <0.1%.

**Table 5:** EO constituents (%) of unpacked (control) lemon verbena in storage duration

No.	Compound	RI^a^	Storage duration (month)				
			0	2	4	6	8
1	α‐pinene	938	0.4	0.7	0.4	t	t
2	Camphene	953	t	t	0.3	0.3	0.9
3	Sabinene	981	1.0	1.8	2.1	0.9	2.4
4	Limonene	1028	13.7	16.9	15.4	11.0	20.3
5	1,8‐cineole	1031	13.3	10.4	15.3	13.7	26.3
6	γ‐terpinene	1062	0.8	t	1.3	1.7	2.3
7	Terpinolene	1090	0.6	0.3	1.1	1.2	1.6
8	Transpinocarveol	1140	t	t	0.1	0.5	t
9	cis‐sabinol	1143	t	t	t	t	t
10	Citronellal	1152	0.5	0.7	0.6	0.2	0.5
11	α‐terpineol	1190	1.2	1.3	1.4	2.3	2.2
12	Nerol	1228	0.4	t	t	0.3	t
13	Neral	1238	11.7	13.5	13.8	12.8	8.1
14	Geranial	1267	17.8	20.3	21.6	19.6	13.3
15	Neryl acetate	1360	1.0	0.8	0.9	1.4	0.7
16	α‐copaene	1379	0.6	0.5	0.5	0.5	0.4
17	α‐gurjunene	1410	t	t	0.2	0.2	t
18	E‐caryophyllene	1421	0.5	1.0	1.1	0.8	0.9
19	γ‐elemene	1439	8.1	7.9	7.4	7.9	5.6
20	α‐humulene	1456	1.0	0.6	1.7	1.8	2.1
21	Cubenol	1514	0.4	t	0.5	0.6	0.1
22	Spathulenol	1580	10.2	6.6	4.4	5.1	3.0
23	Globulol	1587	8.1	8.3	5.9	10.3	5.6
24	Epi‐α‐cadinol	1642	1.2	1.2	0.5	0.9	0.4
Monoterpene hydrocarbons			16.5	19.7	20.6	15.1	27.5
Oxygenated monoterpenes			45.9	47	53.7	50.8	51.1
Sesquiterpene hydrocarbons			10.2	10	10.9	11.2	9
Oxygenated sesquiterpenes			19.9	16.1	11.3	16.9	9.1
Total			92.5	92.8	96.5	94	96.7

a RI, retention indices relative to C8–C25 n‐alkanes on the DB‐5 column; t, trace <0.1%.

Additionally, our results showed that α‐pinene was eliminated gradually during storage in all packaging methods, whereas camphene appeared from the fourth month and then increased. On the other hand, camphene, which could not be detected in the early stages of storage, was only detected at 4 months of storage in all packaging methods (Tables 2, 3, 4 and 5). Therefore, camphene may be a useful marker for the indication of storage duration of *L. citriodora*. Similar results were observed by Topuz and Ozdemir (2004). They reported isodihydrocapsaicin can be used to identify paprika (*Capsicum annuum* L.) which had been stored for longer than 6 months because this compound was only detected from 6 months of storage onward.

## 4 Conclusion

The results showed that the packaging of *L. citriodora* leaves with nitrogen preserved the highest EO content at the end of 8 months of storage. Although vacuum packed leaves preserved the highest amount of citral during storage, the highest amount of limonene and desired contents of citral and 1,8‐cineole were found in leaves packed with nitrogen. This study also showed camphene may be a useful marker for the indication of storage duration of *L. citriodora*.
